# Identifying stationary microbial interaction networks based on irregularly spaced longitudinal 16S rRNA gene sequencing data

**DOI:** 10.3389/frmbi.2024.1366948

**Published:** 2024-06-03

**Authors:** Jie Zhou, Jiang Gui, Weston D. Viles, Haobin Chen, Siting Li, Juliette C. Madan, Modupe O. Coker, Anne G. Hoen

**Affiliations:** ^1^ Department of Biomedical Data Science, Geisel School of Medicine, Dartmouth College, Hanover, NH, United States; ^2^ Khoury College of Computer Science, Northeastern University, Portland, ME, United States; ^3^ Department of Epidemiology, Geisel School of Medicine, Dartmouth College, Hanover, NH, United States; ^4^ School of Dental Medicine, University of Rutgers, Newark, NJ, United States

**Keywords:** Gaussian graphical model, microbial interaction network, EM algorithm, longitudinal data, relative abundance

## Abstract

**Introduction:**

The microbial interactions within the human microbiome are complex, and few methods are available to identify these interactions within a longitudinal microbial abundance framework. Existing methods typically impose restrictive constraints, such as requiring long sequences and equal spacing, on the data format which in many cases are violated.

**Methods:**

To identify microbial interaction networks (MINs) with general longitudinal data settings, we propose a stationary Gaussian graphical model (SGGM) based on 16S rRNA gene sequencing data. In the SGGM, data can be arbitrarily spaced, and there are no restrictions on the length of data sequences from a single subject. Based on the SGGM, EM -type algorithms are devised to compute the *L*1-penalized maximum likelihood estimate of MINs. The algorithms employ the classical graphical LASSO algorithm as the building block and can be implemented efficiently.

**Results:**

Extensive simulation studies show that the proposed algorithms can significantly outperform the conventional algorithms if the correlations among the longitudinal data are reasonably high. When the assumptions in the SGGM areviolated, e.g., zero inflation or data from heterogeneous microbial communities, the proposed algorithms still demonstrate robustness and perform better than the other existing algorithms. The algorithms are applied to a 16S rRNA gene sequencing data set from patients with cystic fibrosis. The results demonstrate strong evidence of an association between the MINs and the phylogenetic tree, indicating that the genetically related taxa tend to have more/stronger interactions. These results strengthen the existing findings in literature.

**Discussion:**

The proposed algorithms can potentially be used to explore the network structure in genome, metabolome etc. as well.

## 1 Introduction

Microorganisms thrive in communities in large numbers. They interact with their host and with one another in various ways, such as commensalism, synergism, competition, parasitism, and predation. This complex set of interactions can be depicted in the form of microbial interaction networks (MINs) (Faust and Raes, 2012). Traditionally, such interactions have been inferred using culture-based methods, which can only accommodate a small number of microbial strains (Gause, 1934; Staley and Konopka, 1985; Harcombe, 2010). Since most microbes cannot be cultivated, the estimated interactions under laboratory conditions could be misleading. Underpinned by advances in next-generation sequencing (NGS) technologies, a complete microbiome profile can be measured at a relatively low cost, allowing researchers to investigate microbial interactions in situ. However, the complexities of these high-throughput data, such as their high dimensionality, zero inflation, and compositional nature, pose substantial challenges to identifying MINs (Faust and Raes, 2012). Currently, the primary way to infer MINs is the pairwise method, in which the cooccurrence or mutual exclusion pattern of two species is compared using measures such as Pearson or Spearman correlation (Qin et al., 2010; Zhou et al., 2010; Arumugam et al., 2011; Barberan et al., 2012). An emerging method is based on conditional independence, i.e., the conditional joint distribution of two taxa given all the other microbiome members. Conditional independence is conceptually superior to the pairwise method since it removes the effects of all the other taxa when measuring the relationship between the two taxa of interest (Kurtz et al., 2015; Chen et al., 2017; Viles et al., 2021). Furthermore, if the data follow a normal distribution, then the precision matrix, i.e., the inverse of the covariance matrix, directly reflects the conditional independence relationship among microbes. With such an appealing interpretation, precision matrices have become the ideal tools for exploring the structure of MINs (Fang et al., 2017; Yoon et al., 2019; Yuan et al., 2019; Jiang et al., 2020; Tian et al., 2023).

In particular, the authors in (Kurtz et al., 2015) proposed a conditional independence-based pipeline named SParse InversE Covariance Estimation for Ecological Association Inference (SPIEC-EASI) to estimate MINs. In SPIEC-EASI, *L*1-penalized maximum likelihood estimation of the precision matrix is employed to identify high-dimensional MINs. Mathematically, the *L*1-penalized maximum likelihood estimation of the precision matrix has been studied extensively in the literature (Yuan and Lin, 2007; Avella-Medina et al., 2018; Wang and Jiang, 2020). Algorithms have been proposed to compute such estimates, e.g., graphical LASSO (Friedman et al., 2008; Friedman et al., 2019) and the neighborhood method (Meinshansen and Bühlmann, 2006). In SPIEC-EASI, graphical LASSO computes the precision matrix recursively based on the coordinate descent algorithm. In contrast, the neighborhood method computes the neighborhood of each node and then combines these neighborhoods to form an estimate of the network. However, a prerequisite of SPIEC-EASI is that the data should be independent. Although independence is a reasonable assumption if the data are cross-sectional, in many other cases, data sets are longitudinal, in which multiple observations are made on the same subject. In such studies, the observations from the same subject are typically correlated and violate the assumption of SPIEC-EASI. There have been studies to estimate the network from the correlated data. The time series models, e.g., vector autoregression, have been employed to address the correlation between observations within the same cluster (Bach and Jordan, 2004; Qiu et al., 2016; Chen et al., 2017; Epskamp et al., 2018; He et al., 2022). In particular, the authors in (He et al., 2022) used autoregression in the proposed ARZIMM model to characterize the longitudinal absolute abundance data for the microbiome study. However, time series methods require the data for each subject to be long enough and equally spaced, which is not usually satisfied in reality. Functional data analysis has also been used to decipher the conditional correlation for high-dimensional data (Zhu et al., 2016; Li and Solea, 2018; Qiao et al., 2019; Solea and Li, 2020). For example, for electroencephalogram (EEG) data (Qiao et al., 2019), proposed a functional graphical model to estimate a network of brain reactions, and (Solea and Li, 2020) proposed the copula Gaussian graphical model for a network of functional magnetic resonance imaging (fMRI) data. Functional data analysis based methods require the data to be densely spaced and the sample size to be large. The requirements inherited in these existing methods are often violated for longitudinal data sets in human microbiome studies.

In this paper, we consider the estimation of MINs from irregularly spaced longitudinal 16S rRNA gene sequencing data. The SPIEC-EASI pipeline can be seen as a special case of the proposed algorithms. The inferences are considered under three conditions. In the first condition, we assume that all subjects share an autocorrelation parameter *τ* during the trial. For this case, we propose a model named the homogeneous SGGM to characterize MINs. For the homogeneous SGGM, a recursive graphical LASSO algorithm is proposed to compute the *L*1-penalized maximum likelihood estimate (MLE) of the network. In the second condition, the homogeneous SGGM is extended to the heterogeneous SGGM, allowing different subjects to have their own autocorrelation parameter. For the heterogeneous SGGM, an expectation-maximization (EM)-type algorithm is devised to compute the *L*1-penalized MLE of the network. In the third condition, the autocorrelation parameters are further allowed to depend on covariates such as sex and race. We show how the algorithm in condition two can be adapted to accommodate the extension. Extensive simulation studies are conducted to compare the proposed algorithms with existing algorithms, including the SPIEC-EASI pipeline and the GGMselect algorithm family (Giraud et al., 2012). The comparisons are conducted under different scenarios, aiming to investigate the robustness of the algorithms to violations of the assumptions of the SGGM. This is necessary since the 16S rRNA gene sequencing data are highly irregular and may fail to exactly satisfy the premises of the proposed models and algorithms. For all the scenarios considered, the proposed algorithms exhibit better performance for network selection than that of other existing algorithms.

In the final part, the proposed models are employed to study a longitudinal gut microbiome data set from a cohort with cystic fibrosis in New Hampshire (Madan et al., 2012). To validate the proposed algorithms, with the estimated MINs, we measure the correlation between the estimated MINs and the corresponding phylogenetic tree. A permutation test is proposed to determine the significance of such a correlation. The results demonstrate strong evidence for the positive correlation between the MINs and the phylogenetic tree, indicating that genetically related taxa also tend to have more/stronger interactions. These findings strengthen the discoveries that have been reported in other studies (Chaffron et al., 2010; Eiler et al., 2012) and provide an empirical basis for using phylogenetic trees as a tool to explore microbial interactions in future studies (Chung et al., 2022).

The paper is organized as follows. In the *Materials and methods* section, we introduce stationary Gaussian graphical models (SGGMs) and three related inference algorithms. In the *Results* section, we compare the performance of the proposed algorithms with that of the conventional methods under different scenarios and demonstrate the superiority of the proposed algorithm. We then considered the gut microbiome of subjects with cystic fibrosis. The homogeneous version of the proposed algorithm is employed to identify the MINs of the microbiome. The plausibility of the estimated MINs is discussed. The *Discussion* section includes a brief review of the models.

## 2 Materials and methods

### 2.1 Data generation process

Let $y_{it_{ik}}={\left(y_{it_{ik1}},\cdot \cdot \cdot ,y_{it_{ikp}}\right)}^{T}$ denote observations of some transformed abundance data of a microbiome with *p* taxa from subject *i* at time *t_k_* (1 ≤ *i* ≤ *m*, 1 ≤ *k* ≤ *n_i_*) so that*k*it is appropriate to assume that $y_{it_{ik}}∼N_{p}\left(µ,\Sigma \right)$ , where $µ=E\left(y_{it_{ik}}\right)$ and $\Sigma =Var\left(y_{it_{ik}}\right)$ . The precision matrix is defined as Ω = Σ^−1^. Then, the *n_i_p* vector ${\text{y}}_{i}={\left({\text{y}}_{i1}^{T},\cdots ,{\text{y}}_{in_{i}}^{T}\right)}^{T}$ represents all the observations on subject *i*, and vector **y** $={\left({\text{y}}_{1}^{T},\cdots ,{\text{y}}_{m}^{T}\right)}^{T}$ represents the observations on all the *m* subjects with $n=\sum_{i=1}^{m}n_{i}$ . For the correlations between the observations, we assume that the observations from different subjects are independent, i.e., $cov\left(y_{i_{1}t_{ik_{1}}},y_{i_{2}t_{ik_{2}}}\right)=0_{p\times p}$ for $i_{1}\neq i_{2},k_{1}\geq 1,k_{2}\geq 1$ . For observations from the 1 same subject, we assume $cov\left(y_{it_{ik_{1}}},y_{it_{ik_{2}}}\right)=DH_{ik_{1}k_{2}}D$ where *D* = diag(*σ*_1_,···,*σ_p_*) with $\sigma_{1}^{2},\cdots ,\sigma_{p}^{2}$ diagonal elements of Σ, while $H_{ik_{1}}{\;}_{k_{2}}$ is the correlation matrix between $Y_{it_{ik_{1}}}$ and $Y_{it_{ik_{2}}}$ for which the following form is assumed:


(1) $$H_{ik_{1}k_{2}}=\Phi_{ik_{1}k_{2}}⊙R$$

The symbol in (Equation 1) stands for the Hadamard product of matrices $\Phi_{ik_{1}k_{2}}$ and *R*. Here, *R* is the correlation matrix with respect to covariance matrix Σ, while matrix $\Phi_{ik_{1}k_{2}}={\left(\phi_{ik_{1}k_{2}}\right)}_{p\times p}$ defines the dampening rates at which the components of $H_{ik_{1}k_{2}}$ decrease as time goes from $t_{ik_{1}}$ to $t_{ik_{2}}$ . For example, ${\left(\Phi_{ik_{1}k_{2}}\right)}_{12}$ is the dampening rate of correlation $cor\left(Y_{it_{ik_{1}}1},Y_{it_{ik_{1}}2}\right)$ to correlation $cor\left(Y_{it_{ik_{1}}1},Y_{it_{ik_{2}}2}\right)$ . Theoretically, dampening rates can vary from taxon to taxon and depend on the time points as long as the resulting matrix *H_ik_*_1_ *k*_2_ is positive definite. However, in this paper, for subject *i*, we assume that the components of $H_{ik_{1}k_{2}}$ have the same dampening rate. Furthermore, they depend on time points $\left(t_{ik_{1}},t_{ik_{2}}\right)$ only through the distance between $t_{ik_{1}}$ to $t_{ik_{2}}$ , i.e., $\phi_{ik_{1}k_{2}}=g_{i}\left(\left|t_{ik_{1}}-t_{ik_{2}}\right|\right)$ for some decreasing function 0 ≤ *g_i_* (·) ≤ 1. Motivated by studies on the longitudinal regression model (Diggle et al., 2002), we assume function *g_i_* (·) has the form of $\exp\left(-\tau_{i}{\left|t_{ik_{1}}-t_{ik_{2}}\right|}^{p}\right)$ . For *p* **=** 0, we have $\phi_{ik_{1}k_{2}}=\exp\left(-\tau_{i}\right)$ , which is referred to as the uniform correlation and can be used to model the spatial correlation. For example, specimens may be collected at different body sites from the same subjects, for which the uniform correlation seems to be a reasonable assumption. On the other hand, the cases of *p >* 0 can be used to model the irregularly spaced temporal correlation, which typically decreases as the time span $\left|t_{ik_{1}}-t_{ik_{2}}\right|$ increases. In particular, functions $\exp\left(\tau_{i}\left|t_{ik_{1}}-t_{k_{2}}\right|\right)$ and $\exp\left(\tau_{i}{\left|t_{ik_{1}}-t_{ik_{2}}\right|}^{2}\right)$ have been used in the marginal regression model for low-dimensional longitudinal data. Here, the parameters *τ_i_* s, which are referred to as autocorrelation parameters, measure the dampening rates that are shared by all the components of $y_{it},\left(i=1,\cdots ,m\right)$ .

Without loss of generality, we always employ the correlation function $\exp\left(\tau_{i}\left|t_{ik_{1}}-t_{ik_{2}}\right|\right)$ in the following and assume that the observations have been centered so that *μ* **=** 0. Let Σ*_i_* denote the covariance matrix of the observation vector ***y*** *_i_*. The density function of *y* is then given by


(2) $$\begin{array}{l} \;f\left(y|\Omega ,\tau \right)=\prod_{i=1}^{m}f_{i}\left(y_{i}|\Sigma_{i},\tau \right),\\ \text{where }f_{i}\left(y_{i}|\Sigma_{i},\tau_{i}\right)={\left(2\pi \right)}^{-n_{i}p/2}|\Sigma_{i}{|}^{-1/2}\exp\left(-y_{i}^{T}\sum_{i}^{-1}y_{i}/2\right)\text{with}\\ \;\sum_{i}=\left(\begin{array}{cccc} \;\Omega^{-1} & e^{-\tau_{i}|t_{i1}-t_{i2}|}\Omega^{-1} & \cdots & e^{-\tau_{i}|t_{i1}-t_{in_{1}}|}\Omega^{-1}\\ e^{-\tau_{i}|t_{i2}-t_{i1}|}\Omega^{-1} & \;\Omega^{-1} & \cdots & e^{-\tau_{i}|t_{i2}-t_{in_{2}}|}\Omega^{-1}\\ \;\vdots & \;\vdots & \;\vdots & \;\vdots \\ e^{-\tau_{i}|t_{in_{i}}-t_{i1}|}\Omega^{-1} & e^{-\tau_{i}|t_{in_{i}}-t_{i2}|}\Omega^{-1} & \cdots & \;\Omega^{-1} \end{array}\right) \end{array}$$

Since the number of unknown parameters in Ω is much larger than the sample size in the context of the gut microbiome, the maximum likelihood estimate of Ω is unidentifiable, and sparsity is typically assumed in the literature. To this end, penalized maximum likelihood estimation (MLE) is usually adopted, e.g., the SPIEC-EASI model in (Kurtz et al., 2015). The SPIEC-EASI pipeline employes the *L*_1_-penalty to achieve the sparsity of Ω for cross-sectional observations. Here, we adopt the same strategy for longitudinal data and use the minimizer of the following *L*_1_-penalized negative log-likelihood function as the estimate of network Ω


(3) $$\left(\hat{Ω},\hat{\tau }\right)=\arg\;\underset{Ω,\tau }{\min}\left\{-2\log\left(f\left(y|Ω,\tau \right)\right)+n\lambda {\left|Ω\right|}_{1}\right\}$$

We refer to model (Equations 2, 3) the stationary Gaussian graphical model (SGGM). Here, stationarity stems from the fact that the same network Ω is shared by all the subjects and at all time points. If the data are independent observations, then (3) can be solved by the graphical LASSO algorithm (Friedman et al., 2008) or the neighborhood method (Meinshansen and Bühlmann, 2006), and SGGM is just reduced to the SPIEC-EASI model. However, since the data are longitudinal and can correlate to each other, the performance of the SPIEC-EASI pipeline is not guaranteed when solving (3).

Notably, in model (2, 3), we assume the same correlation dampening rate *τ_i_* for all the taxa in the microbiome of subject *i*. This assumption is motivated by the characteristics of the gut microbiome, where the taxa are typically influenced by the same perturbation sources, such as diet change and disease development. However, even if this assumption is violated and different taxa have different dampening rates, model (2, 3) can still be used as a working model for identifying the network structure, in which *τ_i_* can be regarded as the mean dampening rate of the whole microbiome. In such cases, models (2, 3) still outperform the SPIEC-EASI pipeline, and the latter ignores the correlation structure of longitudinal data. We demonstrate this point through simulation studies in Section 3.1.

In the following sections, we propose three algorithms to identify the network Ω in (3) based on different dampening rate *τ_i_* models. An algorithm for the homogeneous SGGM is first considered, and two extensions are proposed that allow the algorithms to deal with cohorts of heterogeneous subjects. These algorithms integrate the graphical LASSO algorithm with other algorithms, e.g., the EM algorithm, to find the penalized maximum likelihood estimator of Ω.

### 2.2 Homogeneous SGGM

In this section, we consider identifying Ω under the assumption $\tau_{1}=\cdots =\tau_{m}=\tau$ .

Thus, it is assumed that correlations between observations at different time points dampen at the same rate for each subject in the cohort. From the density function (2), the log-likelihood function for $y={\left(y_{1}^{T},\cdots y_{m}^{T}\right)}^{T}$ is given by


(4) $$l_{n}\left(\Sigma ,\tau |y\right)=-\frac{1}{2}\sum_{i=1}^{m}\left(p\log\left(\left|\Phi_{i}\right|\right)+n_{i}\log\left(\left|\Sigma \right|\right)+y_{i}^{T}{\left(\Phi_{i}⊗\Sigma \right)}^{-1}y_{i}\right)$$

up to a constant. Here, ⊗ stands for the Kronecker product. We use the formulas $\Sigma_{i}=\;\Phi_{i}⊗\Sigma$ and $\left|\Sigma_{i}\right|={\left|\Phi_{i}\right|}^{p}{\left|\Sigma \right|}^{n_{i}}$ . Note that with formula ${\left(\Phi_{i}⊗\Sigma \right)}^{-1}=\Phi_{i}^{-1}⊗\Sigma^{-1}$ , the last term in (Equation 4) can be rewritten as


(5) $$\begin{array}{l} y_{i}^{T}{\left(\Phi_{i}⊗\Sigma \right)}^{-1}y_{i}=y_{i}^{T}\left(\Phi_{i}^{-1}⊗\Sigma^{-1}\right)y_{i}=\sum_{j=1}^{n_{i}}\sum_{k=1}^{n_{i}}\phi_{ijk}^{-}y_{it_{ij}}^{T}\Omega y_{it_{ik}}\\ \;=\sum_{j=1}^{n_{i}}\sum_{k=1}^{n_{i}}\text{tr}\left(\phi_{ijk}^{-}y_{it_{ik}}y_{it_{ij}}^{T}\Omega \right)=\text{tr}\left(\left(\sum_{j=1}^{n_{i}}\sum_{k=1}^{n_{i}}\phi_{ijk}^{-}y_{it_{ik}}y_{it_{ij}}^{T}\right)\Omega \right)\\ \;≜\text{tr}S_{i}\left(\tau \right)\Omega , \end{array}$$

where $S_{i}\left(\tau \right)=\sum_{j=1}^{n_{i}}\sum_{k=1}^{n_{i}}\phi_{ijk}^{-}y_{it_{k}}y_{it_{ij}}^{T}$ and $\Phi_{i}^{-1}=\left(\phi_{ijk}^{-}\right)n_{i}\times n_{i}$ . By substituting (Equation 5) into (4), we have


(6) $$l_{n}\left(\Omega ,\tau |y\right)=-\frac{1}{2}\left\{\sum_{i=1}^{m}p\log\left(\left|\Phi_{i}\right|\right)-n\log\left(\left|\Omega \right|\right)+n\mathrm{tr}\left(\bar{S}\left(\tau \right)\Omega \right)\right\}$$

where $n=\sum_{i=1}^{m}n_{i},\bar{S}\left(\tau \right)=\frac{1}{n}\sum_{i=1}^{m}S_{i}\left(\tau \right)$ . Here, we use $\bar{S}\left(\tau \right)$ to emphasize that matrix $\bar{S}$ is a function of unknown parameter *τ*. With (Equation 6) in hand, the sparse network can be achieved by minimization the *L*_1_-penalized negative log-likelihood function (3), i.e.,


(7) $$\underset{\Omega ,\tau }{\min}\left\{-2l_{n}\left(\Omega ,\tau |y\right)+n\lambda {\left|\Omega \right|}_{1}\right\}$$

for given tuning parameter λ > 0. The minimization problem (Equation 7) can be solved through a block coordinate descent procedure. First note that for a given *τ*, the solution of Ω can be obtained through the following minimization:


(8) $$\underset{\Omega }{\min}\left\{-\log\left|\Omega \right|+tr\left(\bar{S}\left(\tau \right)\Omega \right)+\lambda {\left|\Omega \right|}_{1}\right\}$$

which has the same form as the GGM for independent data when the empirical covariance matrix is given by $\bar{S}\left(\tau \right)$ . Consequently, the graphical LASSO algorithm can be used to compute the sparse estimate of Ω in (Equation 8). On the other hand, given Ω, the minimization of (7) with respect to τ does not involve any *L*_1_ penalty term and consequently can be carried out through the maximization of the likelihood function (6) with respect to τ. The conventional Newton algorithm can be used in this step. This process continues until convergence is achieved. This algorithm will be referred to as homogeneous longitudinal graphical LASSO (LGLASSO), for which the details are summarized in the following table.

**Algorithm 1:** Identify the network based on the homogeneous SGGM.

1: **procedure** Given Initial Value ** *τ* _0_ ** And **Ω_0_ **, Tuning Parameter λ And Error Tolerance ** *e >* 0**: 2: With *τ* **=** *τ* _0_, solve optimization problem (8) with respect to Ω using graphical ** **LASSO and let $\hat{\Omega }$ be the resulting estimate of **Ω**. 3: With $\Omega =\hat{\Omega }$ , solve optimization problem (6) with respect to *τ*. Let $\hat{\tau }$ be the resulting estimate of *τ*. 4: ** if** $\left|\tau_{0}-\hat{\tau }\right|<e$ and $|\left(\Omega_{0}-\hat{\Omega }\right)ij|\;<\;e$ **for 1 ≤ *i* ≤ *j* ≤ *p* then** 5:** **Stop and output $\left(\hat{\tau },\;\hat{\Omega }\right)$ . 6: ** else** 7:** **Let $\tau_{0}=\hat{\tau },\Omega_{0}=\hat{\Omega }$ , return to Step 2.

### 2.3 Heterogeneous SGGM

In the homogeneous SGGM, we assume that a single correlation parameter *τ* applies to all the subjects. In real data analysis, this parameter may vary across subjects. In this section, we consider network identification without assuming $\tau_{1}=\cdots =\tau_{m}$ . Instead, we assume that the parameters *τ_i_*’s are independent random variables from the exponential distribution $\tau_{i}∼\exp\left(\alpha \right)$ . Consequently, the joint density function for ${\left\{y_{i},\tau_{i}\right\}}_{i=1}^{m}$ is


(9) $$\prod_{i=1}^{m}f_{i}\left(y_{i}|\Sigma ,\tau_{i}\right)\alpha \exp\left(-\alpha \tau_{i}\right)$$

from which the likelihood function for Σ and α is given by


(10) $$L_{n}\left(\Omega ,\alpha |y\right)=\int_{\tau_{1},\cdots ,\tau_{m}}\prod_{i=1}^{m}f_{i}\left(y_{i}|\Sigma ,\tau_{i}\right)\alpha_{i}\exp\left(-\alpha \tau_{i}\right)d\tau_{1}\ldots \tau_{m}$$

With (Equations 9, 10) in hand, the sparse estimate for the network can then be obtained by minimizing the following *L*_1_-penalized negative log-likelihood function:


(11) $$\underset{\Omega ,\alpha }{\min}\left\{-2l_{n}\left(\Omega ,\alpha \right)+n\lambda {\left|\Omega \right|}_{1}\right\}$$

where $l_{n}\left(\Omega ,\alpha \right)=\log\left(L_{n}\left(\Omega ,\alpha |y\right)\right)$ . Since no explicit form for $l_{n}\left(\Omega ,\alpha \right)$ is available, the expectation-maximization (EM) algorithm is proposed here to find the solution to (11) (Dempster et al., 1977). Since we are considering the negative log-likelihood function in (11), the maximization in the EM algorithm will be replaced by the minimization. The correlation parameters $\tau =\left(\tau_{1},\cdots ,\tau_{m}\right)$ will be taken as the so-called missing data. Recall in the first step of the EM algorithm that the conditional distribution of missing data *τ* given *y*, Σ = Σ_0_ *, α* = *α*_0_ has to be derived from (2) and (9) as follows:


(12) $$\begin{array}{l} \;g\left(\tau |y,\Sigma_{0},\alpha_{0}\right)=\prod_{i=1}^{m}g\left(\tau_{i}|y_{i},\Sigma_{0},\alpha_{0}\right)\\ \propto \prod_{i=1}^{m}{\left|\Phi_{i}\right|}^{-p/2}\exp\left(-y_{i}^{T}\left(\Phi_{i}^{-1}⊗\Sigma_{0}^{-1}\right)y_{i}/2\right)\exp\left(-\alpha_{0}\tau_{i}\right) \end{array}$$

For (12), the expectation of the complete log-likelihood function for (*y, τ*) to (12) has to be computed. Given the joint density function (9) of (*y, τ*), the expectation of its logarithmic transformation can be shown to be


(13) $$\begin{array}{l} Q\left(\Omega ,\alpha |\Omega_{0},\alpha_{0}\right)=\sum_{i=1}^{m}pE_{g}\left\{\log\left(\left|\Phi_{i}\right|\right)\right\}-n\log\left(\left|\Omega \right|\right)+ntr\left(S^{\left(0\right)}\Omega \right)\\ \;-2m\log\left(\alpha \right)+2\alpha \sum_{i=1}^{m}E_{g}\tau_{i}+n\lambda {\left|\Omega \right|}_{1} \end{array}$$

where $\bar{S}\left(\tau \right)=\frac{1}{n}\sum_{i=1}^{m}S_{i}\left(\tau_{i}\right),S^{\left(0\right)}=E_{g}\bar{S}\left(\tau \right)=\frac{1}{n}\sum_{i=1}^{m}E_{g}S_{i}\left(\tau_{i}\right)$ in which $S_{i}\left(\cdot \right)$ is defined in the previous section. In the second step of the EM algorithm, the minimum point of the *Q* function in (13) has to be computed. This, again, is implemented through a block coordinate descent algorithm. First, for fixed Ω, it is straightforward to show that the minimum of the *Q* function with respect to *α* is attained at


(14) $$\hat{\alpha }=\frac{1}{\frac{1}{m}\sum_{i=1}^{m}E_{g}\tau_{i}},$$

i.e., the reciprocal of the sample mean of the conditional expectation of *τ_i_* with respect to density (12). Then, for a given *α* in (Equation 14), the minimization of (13) with respect to Ω is equivalent to


(15) $$\underset{\Omega }{\min}\left\{-\log\left|\Omega \right|+tr\left(S^{\left(0\right)}\Omega \right)+\lambda {\left|\Omega \right|}_{1}\right\}$$

which can be solved through the graphical LASSO algorithm. The difficult part of this algorithm is to find the expectation $E_{g}\bar{S}\left(\tau \right)$ , which may not have an explicit form given that $\bar{S}\left(\tau \right)$ is a nonlinear function of *τ* and the complex form of density function *g*(*τ*); therefore, this expectation will be computed through a Monte Carlo method. This algorithm will be referred to as the heterogeneous longitudinal graphical LASSO, for which the details are summarized in the following table. Given initial values *α*_0_ and Ω_0_, tuning parameter *λ* and error tolerance *e >* 0, the following algorithm is applied.

### 2.4 Covariate-adjusted SGGM

In the previous section, we assumed that $E\tau_{I}=1/\alpha$ is constant across the subjects. In this section, we further relax this constraint, and *α* can be a function of the covariates. Specifically, we assume $\tau_{i}∼exp\left(\alpha_{i}\right)$ , where *α_i_* has the following form:


(16) $$\alpha_{i}=\exp\left(\alpha_{0}+\alpha_{1}x_{i1}+\cdots +\alpha_{q}x_{iq}\right)=\exp\left(\alpha^{T}x_{i}\right).$$

**Algorithm 2:** Identify the network based on the heterogeneous SGGM.

** 1: procedure** GIVEN INITIAL VALUES ** *α* _0_ ** AND **Ω_0_ **, TUNING PARAMETER λ AND ERROR TOLERANCE ** *e >* 0**: 2: Sample $\tau_{ij}\left(i=1,\cdots ,m\right)$ from the distribution (12) with **Ω = Ω_0_ **; 3: Estimate $E\left(\tau \right),E_{g}S_{i}\left(\tau_{i}\right)$ and $S^{\left(0\right)}$ by $\frac{1}{h}\sum_{j=1}^{h}\tau_{i},\frac{1}{h}\sum_{j=1}^{h}S_{i}\left(\tau_{ij}\right)$ and $\frac{1}{mh}\sum_{i=1}^{m}\sum_{j=1}^{h}S_{i}\left(\tau_{ij}\right)$ respectively 4: Update ** *α* ** by $\hat{\alpha }$ in (14), **Ω** by $\hat{\Omega }$ , the solution to (15), in which ** *E*(*τ*)** and ** *S* ^(0)^ ** are replaced by their estimates in Step 3. 5: **if** $\left|\alpha_{0}-\hat{\alpha }\right|<e$ and ${\left|{\left(\Omega_{0}-\hat{\Omega }\right)}_{ij}\right|}_{1}<\;e$ **then** 6: Stop and output $\left(\hat{\alpha },\hat{\Omega },{\hat{\tau }}_{í},i\;=\;1,\cdots ,m\right)$ . 7:** else** 8: $\alpha_{0}=\hat{\alpha }$ and $\Omega_{0}=\hat{\Omega }$ , return to Step 2.

Here, $x_{i}=\;{\left(1,x_{i1},\cdots ,x_{iq}\right)}^{T}$ represents covariates such as sex and race, and $\alpha ={\left(\alpha_{0},\cdots ,\alpha_{q}\right)}^{T}$ represents unknown parameters. The model (Equations 9–15) and **Algorithm 2** in the previous section can then be revised straightforwardly to accommodate the current regression model (Equation 16). Specifically, first replace the parameter *α* in (Equations 9, 10) by $exp\left(\alpha^{T}x_{i}\right)$ . Then, the conditional distribution of missing data is given by


(17) $$\begin{array}{l} g\left(\tau |y,\Sigma_{0},\alpha_{0}\right)=\prod_{i=1}^{m}g\left(\tau_{i}|y_{i},\Sigma_{0},\alpha_{0}\right)\\ \;\propto \prod_{i=1}^{m}{\left|\Phi_{i}\right|}^{-p/2}\exp\left(-y_{i}^{T}\left(\Phi_{i}^{-1}⊗\Sigma_{0}^{-1}\right)y_{i}/2\right)\exp\left(-\exp\left(\alpha_{0}^{T}x_{i}\right)\tau_{i}\right) \end{array}$$

Based on (Equation 17), the *L*_1_-penalized likelihood estimation of the MIN (11) is then given by


(18) $$\underset{\Omega ,\alpha }{\text{min}}\left\{-2l_{n}\left(\Omega ,\alpha \right)+n\lambda |Ω{|}_{1}\right\}$$

For the same reason, we need to use the EM algorithm to solve the optimization problem (Equation 18). The conditional distribution of missing value $\left\{\tau_{i},i=1,\cdots ,m\right\}$ is given by (12), with *α*_0_ replaced by $\text{exp}\left(\alpha_{0}^{T}x_{i}\right)$ , from which the *Q* function is given by


(19) $$\begin{array}{l} Q\left(\text{Ω},\alpha |{\text{Ω}}_{0},\alpha_{0}\right)=\sum_{i=1}^{m}pE_{g}\left\{\log\left(\left|{\text{Φ}}_{i}\right|\right)\right\}-n\log\left(\left|\text{Ω}\right|\right)+ntr\left(S^{\left(0\right)}\text{Ω}\right)\\ \;-2\sum_{i=1}^{m}\left\{\alpha^{T}x_{i}-\exp\left(\alpha^{T}x_{i}\right)E_{g}\tau_{i}\right\}+n\lambda {\left|\text{Ω}\right|}_{1,} \end{array}$$

Given the penalized *Q* function (Equation 19), the current estimate of the network Ω and parameter *α*, defined as the minimum point of *Q*, can be computed by the block coordinate descent algorithm. However, unlike (14), in the current case, the estimate of *α* does not admit an explicit form. We have to use numerical methods such as Newton algorithms to find the minimizer of the *Q* function (19). Specifically, we solve the following problem by using the BFGS algorithms:


(20) $$\hat{\alpha }=\underset{\alpha }{\min}\left\{\sum_{i=1}^{m}\left(-\alpha^{T}x_{i}+\exp\left(\alpha^{T}x_{i}\right)E_{g}\tau_{i}\right)\right\}$$

**Algorithm 3:** Identify the network based on the covariate-adjusted SGGM.

1: **procedure** GIVEN INITIAL VALUES ** *α* ** _0_ AND **Ω** _0_, TUNING PARAMETER **λ** AND ERROR TOLERANCE ** *e >* 0**: 2: Sample $\tau_{ij}(i=1,\cdots ,m)$ from the distribution (17); 3: Estimate $E\left(\tau \right),E_{g}S_{i}\left(\tau_{i}\right)$ and $S^{\left(0\right)}$ by $\frac{1}{h}\sum_{j=1}^{h}\tau_{i},\frac{1}{h}\sum_{j=1}^{h}S_{i}\left(\tau_{ij}\right)$ and $\frac{1}{mh}\sum_{i=1}^{m}\sum_{j=1}^{h}S_{i}\left(\tau_{ij}\right)$ respectively 4: Update ** *α* ** by $\hat{\alpha }$ the solution to (20), **Ω** by $\hat{\text{Ω}}$ , the solution to (15), in which ** *E*(*τ*)** and ** *S* ** ^(0)^ are replaced by their estimates in Step 3. 5: **if** $\left|\alpha_{0}-\hat{\alpha }\right|\;<\;e$ and ${\left|{\left({\text{Ω}}_{0}-\hat{\text{Ω}}\right)}_{ij}\right|}_{1}\;<\;e$ **then** 6: Stop and output $\left(\hat{\text{α}},\hat{\text{Ω}},{\hat{\tau }}_{í},i=1,\cdots ,m\right)$ . 7: **else** 8: $\alpha_{0}=\hat{\text{α}}$ and ${\text{Ω}}_{0}=\hat{\text{Ω}}$ , return to Step 2.

Using $\hat{\alpha }$ in (Equation 20), the estimate of the MIN Ω is given by the solution of Equation (15) through the graphical LASSO algorithm. The algorithm is summarized in the following.

**Remark:** (1) All three algorithms described above leverage the graphical LASSO algorithm to achieve efficiency even though graphical LASSO itself is devised for independent data. (2) Note that correlation *τ_i_* for subject *i* is a random variable. The forecast of *τ_i_*, ${\hat{\tau }}_{í}$ is given by the expectation of distribution (12), which is one of the outputs in **Algorithms 2** and **3**. (3) The proposed algorithms can generate a solution path for a given sequence of tuning parameters *λ*. To select the optimal network Ω from the candidate networks, model selection criteria can be used, e.g., Akaike information criterion (AIC), Bayesian information criterion (BIC), or cross-validation (CV). In the numerical studies in the next section, we use the extended BIC (EBIC) that is dedicated to the graphical model to select *λ* (Foygel and Drton, 2010).

## 3 Results

### 3.1 Simulation

In this section, we compare the proposed algorithms, which are referred to as longitudinal graphical LASSO (LGLASSO) algorithms, with other existing network selection methods. These methods include graphical LASSO (Friedman et al., 2008; Friedman et al., 2019), neighborhood (Meinshansen and Bühlmann, 2006), GGMselectC01 and GGMselect-LA algorithms (Giraud et al., 2012; Bouvier et al., 2022). The graphical LASSO and neighborhood algorithms have been used in the SPIEC-EASI pipeline in (Kurtz et al., 2015) to select MINs and both are based on the *L*_1_-penalty. On the other hand, the GGMselect algorithm family provides different ways to construct and select the candidate models, e.g., GGMselectC01 employs the estimation procedure in (Wille and Bühlmann, 2006) to construct the candidate models, while GGMselect-LA uses the Fisher random variable to define the criterion for network selection. We demonstrate that the proposed longitudinal graphical LASSO algorithms can outperform these existing algorithms for simulated high-dimensional longitudinal microbiome data.

Let TP,P,FP,N,TN be the numbers of true positive edges, real positive edges, false-positive edges, null edges, and true null edges, respectively. In the following, we use the true/false positive rate (TPR/FPR) to measure the performance of each algorithm. They are defined as TPR **=** TP*/*P, FPR **=** FP*/*N. Also, for the conventional indices sensitivity*/*specificity, we have sensitivity **=** TPR, specificity **=** TN*/*N

The simulations consist of three scenarios. In the first scenario, we consider cases where the data follow the SGGM in Sections 2.2 and 2.3. Recall that the SGGM assumes that all the taxa in the microbiome share a common dampening rate *τ_i_* for subject *i*. We refer to such microbiomes as having a homogeneous community. In the second scenario, we consider the microbiomes that violate such homogeneity, i.e., having heterogeneous communities. In scenario three, we consider the left-censored microbiome data, which aims to test the robustness of the SGGM with respect to zero inflation. The zero-inflation phenomena are widely observed in 16S rRNA gene sequencing experiments and violate the assumptions of the SGGM. Both the homogeneous and heterogeneous versions of LGLASSO in Sections 2.2 and 2.3 are investigated in each scenario. We use the receiver operating characteristic (ROC) curve to show the superiority of our algorithms over the graphical LASSO and the neighborhood algorithms in all scenarios. In the case of the heterogeneous LGLASSO, we also use (TPR,FPR) to compare the performances of the algorithms in which the networks are selected through the extended BIC (EBIC). The methods of GGMselect-C01 and GGNselect-LA have their own model selection approaches for network selection. The EBIC for the Gaussian graphic model is given by


(21) $$\text{EBIC}(G)=-2l_{n}(G)+\left|G\right|\text{log}\left(n\right)+\left|G\right|\text{log}\left(p\right)/T$$

where *l_n_* is the log-likelihood function, *n* is the sample size, *G* is the network of interest, ***|*** *G* ***|*** is the number of edges in *G*, p is the number of nodes, and *T* is the tuning parameter. In (Equation 21), we choose a typical value *T* **=** 2 for model selection.

#### 3.1.1 Scenario 1: homogeneous microbial community

Since homogeneity/heterogeneity involves both microbial community and LGLASSO algorithms, we will use more specific names, homogeneous-subject LGLASSO (heterogenoussubject LGLASSO), to refer to the algorithm to avoid confusion in the following sections.

In a homogeneous microbial community, for each subject, all taxa in the microbiome share a common correlation-dampening rate. Based on whether the subjects share dampening rates, we have the homogeneous-subject LGLASSO in Section 2.2 and heterogeneous-subject LGLASSO in Sections 2.3 and 2.4, respectively. We first consider the former case. Specifically, we consider networks with *p* **=** 80 nodes shared by all *m* **=** 10 subjects. The precision matrix corresponding to this network is generated through the R package *BDgraph* (Mohammadi and Wit, 2019) with an edge density equal to 0.1. For subject *i* (1 ***≤*** *i* ***≤*** *m*), there are *n_i_* observations where *n_i_* follows Poisson distribution with the mean value of 10. The spaces between two consecutive time points *t_ij_* and *t_i_* **_(_** *_j_* **_+_**_1_ **_)_** are generated by max{Δ*_ij_*, 0.5}, where Δ*_ij_* follows a Poisson distribution with a mean value of 1. Then, the data for subject *i* are generated with the real autocorrelation parameter in **Algorithm 1** given by *τ*.

**Figure 1:**
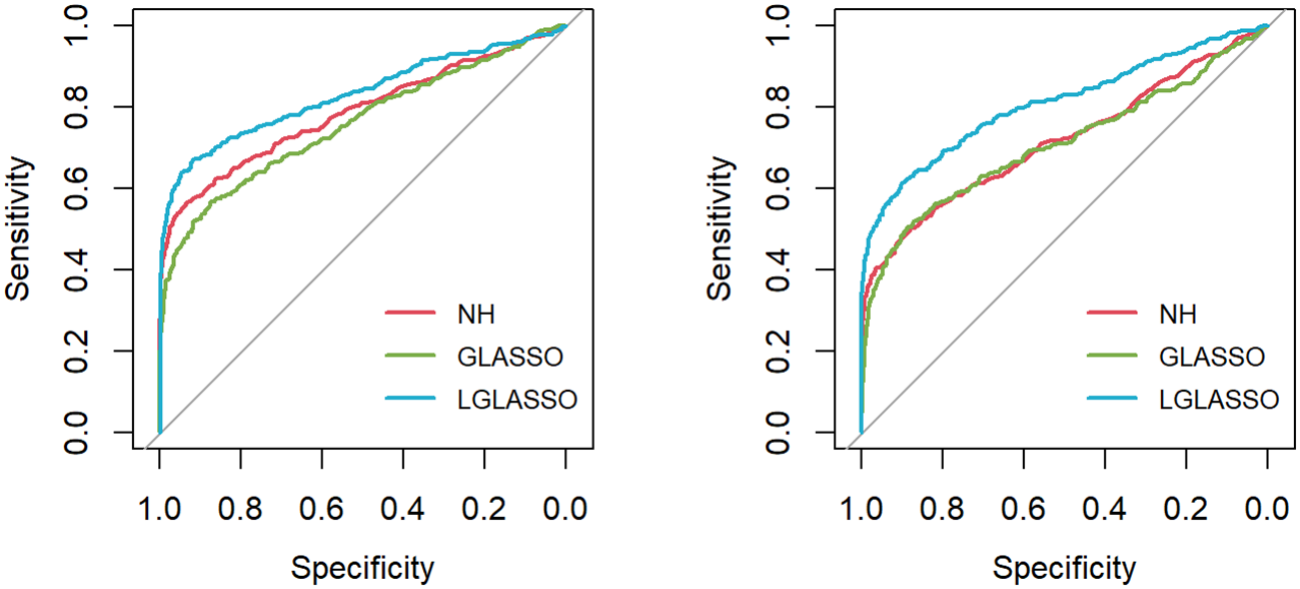
ROC curves for the homogeneous-subject LGLASSO, graphical LASSO, and neighborhood algorithms. The data are generated based on the homogeneous SGGM in Section 2.2. The dampening rates for the left and right plots are *τ* **=** 0.36,0.018, respectively.

First, the homogeneous-subject LGLASSO, graphical LASSO, and neighborhood algorithms are carried out for the simulated data set, from which their respective solution paths are computed. For each path, the connection probability *p_ij_* for any given node pair (*V_i_,V_j_*) is then computed as the proportion of networks that have (*V_i_,V_j_*) connected among all the networks on the path. The processes are replicated 50 times, and the final estimate for *p_ij_* is the average of these 50 estimates of *p_ij_*. With these *p_ij_* (1 ***≤*** *i* ***≤*** *j* ***≤*** 80) in hand, the ROC curve is computed based on the R package *pROC* (Robin et al., 2011) and displayed in **Figure 1** under the two indicated situations *τ* **=** 0.14,0.018. In both cases, the proposed homogeneous-subject LGLASSO algorithm outperforms the graphical LASSO and the neighborhood algorithms. The differences between these ROC curves are more evident for *τ* **=** 0.018 than *τ* **=** 0.14. We interpret this phenomenon as the proposed LGLASSO can better handle the correlated longitudinal data than other algorithms.

**Figure 2:**
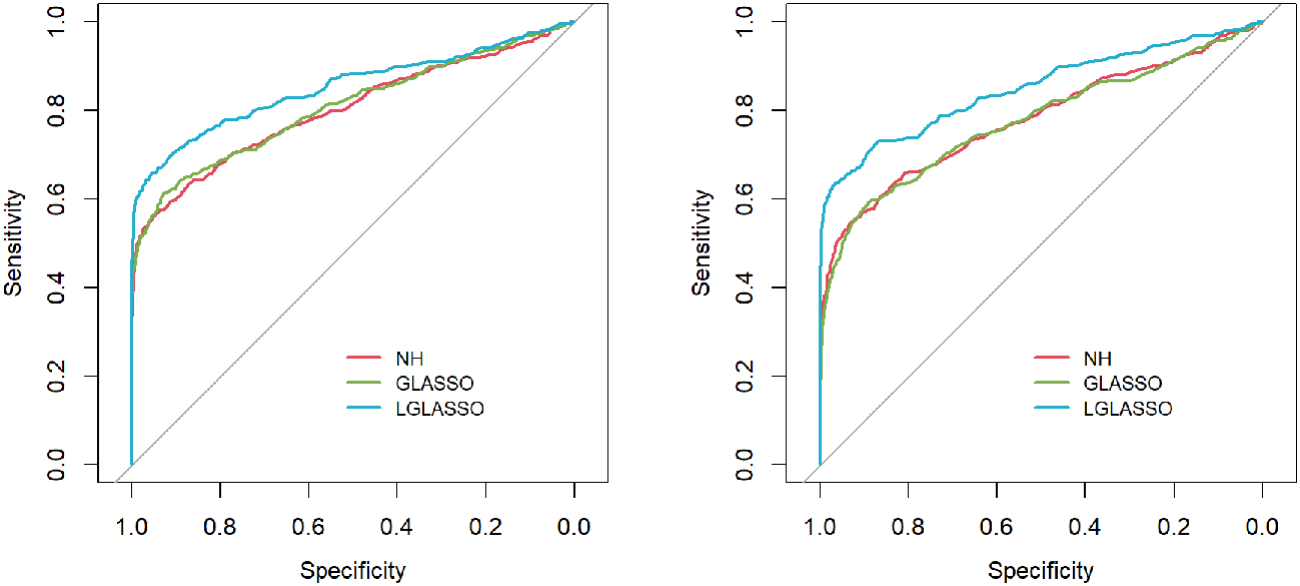
ROC curves for the heterogeneous-subject LGLASSO, graphical LASSO, and neighborhood algorithms. The data are generated based on the heterogeneous SGGM in Section 2.3. The average dampening rates for the left and right plots are *Eτ* **=** 0.14,0.018, respectively.

Next, we consider the heterogeneous-subject LGLASSO and covariate-adjusted LGLASSO algorithms proposed in Sections 2.3 and 2.4, respectively. For ease of exposition, we consider the heterogeneous-subject LGLASSO as a special covariateadjusted LGLASSO in which vector 1 is the only covariate. As in Scenario 1, we still consider a network with *p* **=** 80 nodes and an edge density equal to 0.1. For each subject of *m* **=** 10 subjects, the spaces between consecutive observations are generated similarly. Since the dampening rate *τ* is an exponential random variable in the heterogeneous-subject LGLASSO, we generate random samples as the individual *τ_i_* s from the following two settings, *Eτ* **=** 0.14,0.018. Then with the same replication scheme as above, the ROC curves are computed and plotted in **Figure 2**. Similar to the case of homogeneous-subject LGLASSO, these ROC curves also demonstrate the superiority of heterogeneous-subject LGLASSO over other methods, especially for the higher correlation case *Eτ* **=** 0.018.

We then employ the EBIC (21) to select the optimal model from the solution path. Specifically, two covariates *x*_1_ and *x*_2_ are introduced in which *x*_1_ ∼ *N*(0,1) and *P*(*x*_2 _= 0**) =** *P*(*x*_2 _= 1**) =** 0.5. The three settings for their coefficients (*α*_1_ *, α*_2_) in Equation (16) are (*α*_1_ *, α*_2_ **) = (**0,0), (0.5, 0.5),(1,1) while the intercept is always *α*_0_ = 4. Note that with (*α*_1_ *, α*_2_ **) = (**0,0), the covariates have no effect on the dampening rate, and therefore, the model reduces to the heterogeneous-subject LGLASSO in Section 2.3. For each simulated data set, the pairs (TPR,FPR) can be computed. The process is replicated 50 times and the averages of these 50 (TPR,FPR) are listed in **Table 1**. Note that for GGMselect-C01 and GGMselect-LA listed in **Table 1**, we used their own model selection method instead of the EBIC. From **Table 1**, we can see that the proposed LGLASSO algorithm obtains the highest TPR and lowest FPR in most cases. In other words, with the EBIC as the model selection method, the heterogeneous-subject LGLASSO algorithms still have the best performance among the algorithms considered.

**Table 1:** Performance comparison of heterogeneous-subject LGLASSO (LGLASSO), graphical LASSO (GLASSO), neighborhood (NH) algorithm, GGMselect-C01 (C01), and GGMselect-LA (LA).

			*α* = (0,0)		*α* = (0.5,0.5)		*α* = (1,1)	
			TPR	FPR	TPR	FPR	TPR	FPR
*m* = 10	*E*(*n_i_*) = 5	GLASSO	0.489	0.282	0.508	0.299	0.480	0.311
		NH	0.522	0.297	0.567	0.311	0.541	0.327
		C01	0.138	0.037	0.295	0.126	0.321	0.174
		LA	0.328	0.111	0.369	0.125	0.367	0.151
		LGLASSO	**0.603**	**0.276**	**0.617**	**0.274**	**0.591**	**0.256**
*m* = 10	*E*(*n_i_*) = 10	GLASSO	0.545	0.327	0.551	0.314	0.476	0.318
		NH	0.654	0.394	0.640	0.371	0.568	0.339
		C01	0.528	0.380	0.509	0.320	0.458	0.310
		LA	0.583	0.291	0.549	0.252	0.546	0.289
		LGLASSO	**0.690**	**0.246**	**0.671**	**0.220**	**0.586**	**0.203**
*m* = 10	*E*(*n_i_*) = 20	GLASSO	0.631	0.370	0.576	0.329	0.534	0.316
		NH	0.737	0.506	0.687	0.412	0.644	0.374
		C01	0.852	0.801	0.809	0.744	0.820	0.748
		LA	0.746	0.505	0.730	0.473	0.711	0.448
		LGLASSO	**0.722**	**0.213**	**0.732**	**0.266**	**0.696**	**0.239**

The data are generated based on the heterogeneous SGGM in Section 2.3.

The bold values are the results of the proposed algorithm LGLASSO.

#### 3.1.2 Scenario 2: heterogeneous microbial community

In Section 2.1, we mentioned that the taxa from the same subject are supposed to share the same correlation-dampening rate. In this section, we show by simulation that even if the microbiome fails to satisfy this assumption, the proposed algorithm can still outperform the conventional methods. Specifically, in the heterogeneous microbial community, the taxa have different correlation-dampening rates for each subject. For ease of exposition, we consider a simple situation where the microbiome consists of two subcommunities, A and B, which have different correlation-dampening rates *τ_A_* and *τ_B_*, respectively. Furthermore, we assume that these two communities are independent of each other, i.e., the taxa in community A (B) can correlate with one another; however, they are independent of the taxa in community B (A).

**Figure 3:**
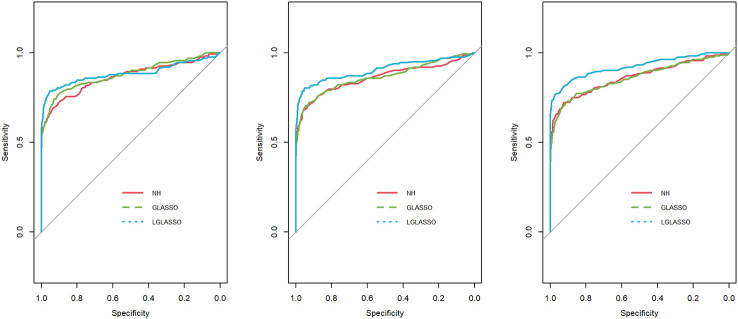
ROC curves for the homogeneous-subject LGLASSO, graphical LASSO, and neighborhood algorithms. The data are generated from homogeneous SGGM with a heterogeneous microbiome. The dampening rates, from left to right, are (*τ_A_,τ_B_***) = (**0.018,0.36), (0.018,0.049), and (0.018,0.018), respectively.

Specifically, for *p* **=** 80 taxa, we assume the first 40 taxa are in community A, and the other 40 taxa are in community B. As in Scenario 1, we first investigate the performance of the homogeneous-subject LGLASSO algorithm. For *m* **=** 10*, En_i_* **=** 10*, τ_A_* **=** 0.018, with a given *τ_B_*, the data for communities A and B are generated by the same method as that in Scenario 1. Since we assume these two communities are independent, the complete data set is simply a combination of the data sets from communities A and B. Three solution paths for the homogeneous-subject GLASSO, graphical LASSO, and neighborhood algorithms are then computed based on the combined data set from which the estimates of the connection probability *p_ij_* s are computed the same way as in Scenario 1. Based on these *p_ij_* s, the ROC curves are plotted in **Figure 3** for the three settings, *τ_B_* **=** 0.36,0.049, and 0.018. Obviously, for heterogenous communities, the proposed homogeneous-subject LGLASSO still outperforms the other two methods, especially when the correlation is higher (i.e., *τ_B_* **=** 0.018)

**Figure 4:**
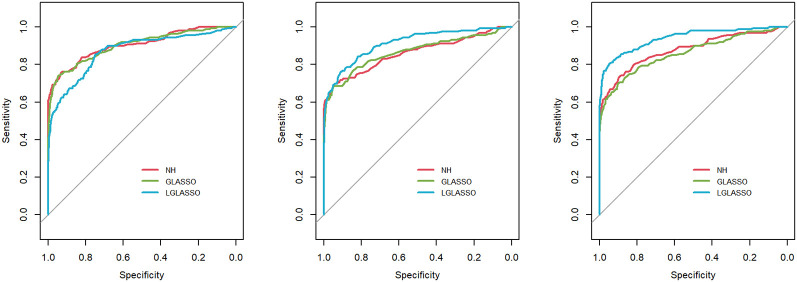
ROC curves for the heterogeneous-subject LGLASSO, graphical LASSO, and neighborhood algorithms. The data are generated from heterogeneous SGGM with a heterogeneous microbiome. The average dampening rates, from left to right, are (*Eτ_iA_, Eτ_iB_***) = (**0.036,0.36), (0.036,0.14), and (0.036,0.049), respectively.

Next, we consider the performance of the heterogeneous-subject LGLASSO. The data are generated from heterogeneous subjects with heterogeneous microbial communities. For ease of exposition, we focus on the heterogeneous-subject model in Section 2.3, and the covariate-adjusted model in Section 2.4 is omitted here. Specifically, for subject *i*, the corresponding microbiome consists of two microbial communities that have correlation dampening rates *τ_iA_* and *τ_iB_* (1 ***≤*** *i* ***≤*** *m*) and satisfy *τ_iA_* ∼ exp(*α*1), *τ_iB_* ∼ exp(*α*_2_). The parameter settings for (*Eτ_iA_, Eτ_iB_*) include the following three cases: (*Eτ_iA_, Eτ_iB_* **) = (**0.036,0.36),(0.036,0.14),(0.036,0.049). For each (*α*_1_ *, α*_2_) pair, 10 random samples are generated for (*τ_iA_,τ_iB_*) from the corresponding exponential distributions, which are used as the real autocorrelation parameters for the 10 subjects. With (*τ_iA_,τ_iB_*), *n_i_* measurements for subject *i* are then generated in the same way as in **Figure 3** with *En_i_* **=** 10. With these data, the heterogeneous-subject LGLASSO, graphical LASSO, and neighborhood algorithms are carried out, and the resulting ROC curves are presented in **Figure 4**. These ROC curves demonstrate that with heterogeneous communities and heterogeneous subjects, the proposed algorithm LGLASSO still outperforms graphical LASSO and neighborhood methods when the correlations between data are reasonably high.

#### 3.1.3 Scenario 3: zero-inflated relative abundances

**Figure 5:**
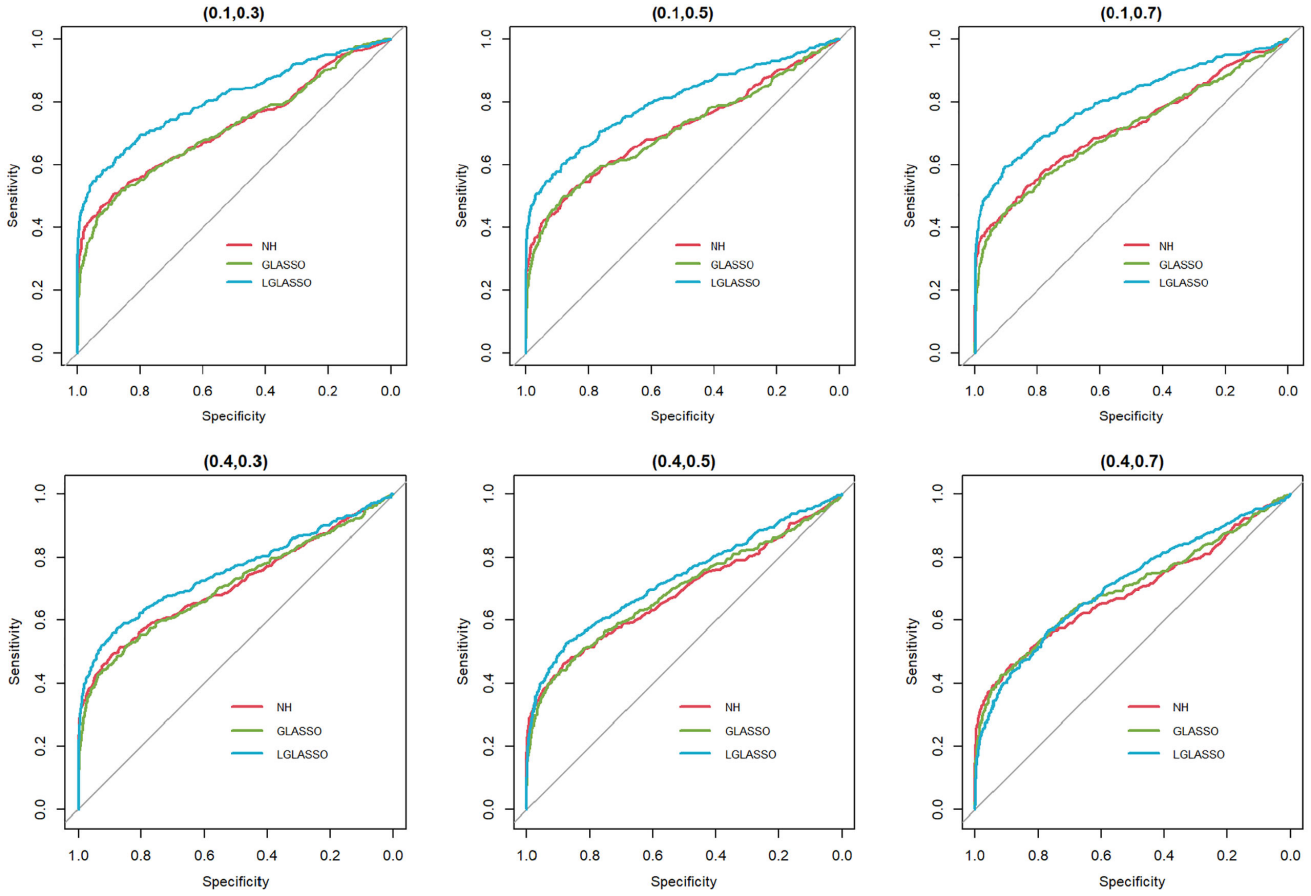
ROC curves for the homogeneous-subject LGLASSO, graphical LASSO, and neighborhood algorithms. The data are generated from a left-censored homogeneous SGGM model. The first number in parentheses is the quantile, and the second is the probability, which are the parameters for data censoring.

**Figure 6:**
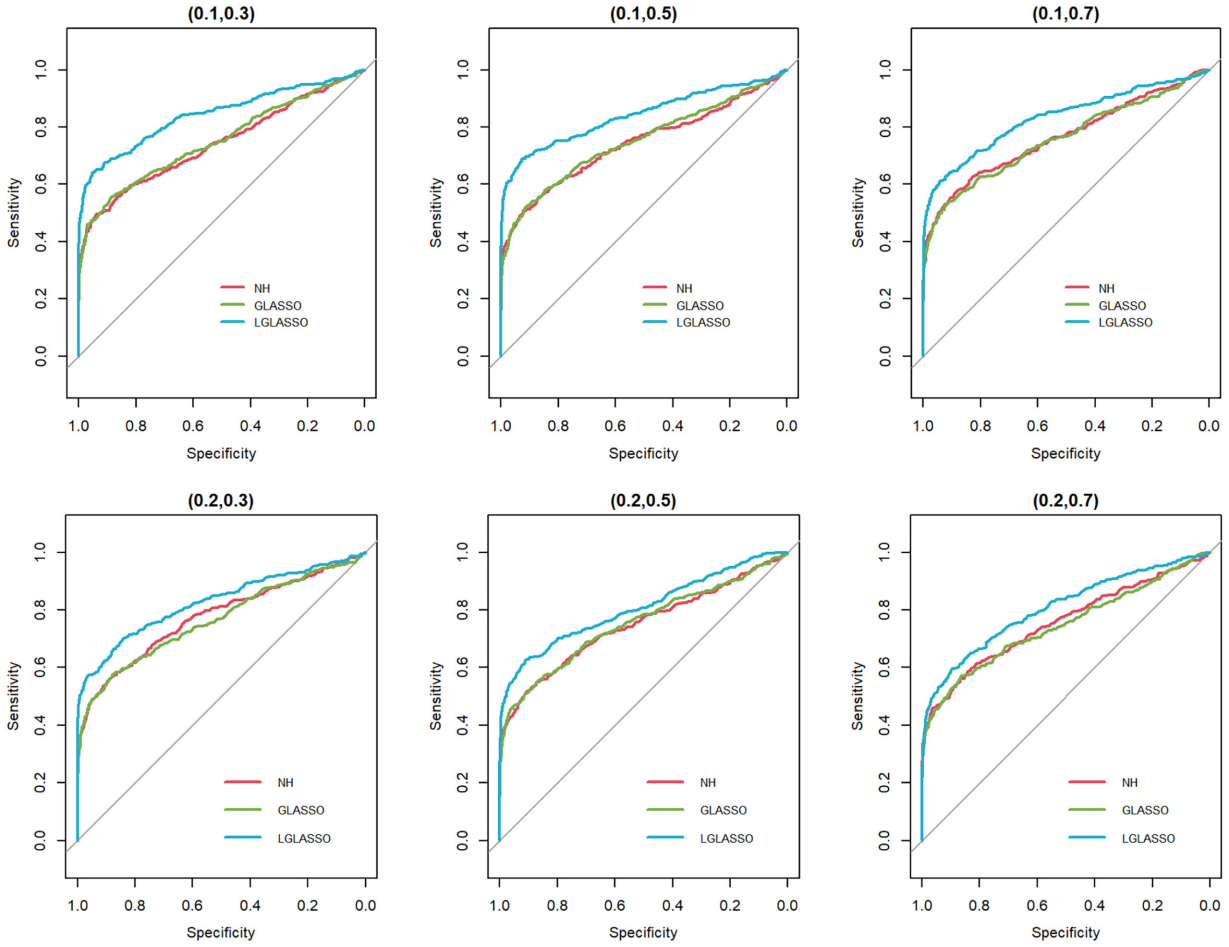
ROC curves for the heterogeneous-subject LGLASSO, graphical LASSO, and neighborhood algorithms. The data are generated from a left-censored heterogeneous SGGM model. The first number in parentheses is the quantile, and the second is the probability, which are the parameters for data censoring.

In this section, we consider the performance of the algorithms when the data generated from the SGGM are left-censored. Left-censored data represent the transformed zero-inflated relative abundance of 16S rRNA gene sequences. An example of such transformations is shown in Section 3.2 in the real data analysis. Here, we investigate the influence of zero inflation on the performance of the proposed algorithms. As in Scenario 1, we consider left-censored homogeneous-subject LGLASSO with *m* **=** 10, *En_i_* **=** 10 (1 ***≤*** *i* ***≤*** *m*) and *τ* **=** 0.018. Under this setting, the data are first generated in the same way as in Scenario 1. Using the generated data, we consider the following censoring scheme: given 0< *q*_1_< 1, for each taxon, all observations with values less than quantile *y_q_*_1_ are replaced by *y_q_*_1_ with a probability of *q*_2_. This censoring scheme is motivated by the observation that the smaller the relative abundance is, the higher the probability is that this taxon is missed by the sequencing experiments. Here, we consider six combinations of (*q*_1_ *, q*_2_), i.e., (0.1,0.3),(0.1,0.5),(0.1,0.7),(0.4,0.3),(0.4,0.5),(0.4,0.7). For each of these combinations, the ROC curves corresponding to the respective solution paths of the homogeneous-subject LGLASSO, graphical LASSO, and the neighborhood algorithms are shown in **Figure 5**. Obviously, even though the proposed homogeneous-subject LGLASSO algorithm outperforms the other algorithms, zero inflation can significantly affect its performance, and the advantage of LGLASSO diminishes when the proportion of zero is high. The same investigations are carried out for the heterogeneous-subject LGLASSO. The procedure is the same as the above homogeneous case except that the dampening rate is random with *Eτ_i_* **=** 0.018 for (1 ***≤*** *i* ***≤*** *m*). The resulting ROC curves are depicted in **Figure 6**, from which we can see a similar pattern as the ones in **Figure 5**.

### 3.2 Gut microbial interaction network and phylogenetic tree

In this section, a longitudinal data set from a cohort of children with cystic fibrosis was investigated using the homogeneous version of the proposed algorithm in Section 2.2. Specifically, stool samples from thirty-eight children were collected from children aged 6 months to 51 months old (Madan et al., 2012). The number of observations from each child ranged from 2 to 17. Each observation consisted of the abundance of 16,383 amplicon sequence variants (ASVs) of the 16S rRNA gene. These sequences were then collapsed to the genus level using the R package *DADA2* (Callahan et al., 2016). The sequences that had no genus-level information were dropped. Then, all the taxa with a proportion of nonzero observations less than 10% were combined, which was referred to as the composite taxon. There were 83 total remaining taxa. The observations of zeros for each of these 83 taxa were replaced by the minimum abundance of that taxon divided by 10. The log-ratio transformation was then carried out to obtain the relative abundance, in which the composite taxon was used as the reference. Similar log-ratio transformations have been used and justified in empirical studies (Kurtz et al., 2015; Greenacre et al., 2021).

**Figure 7:**
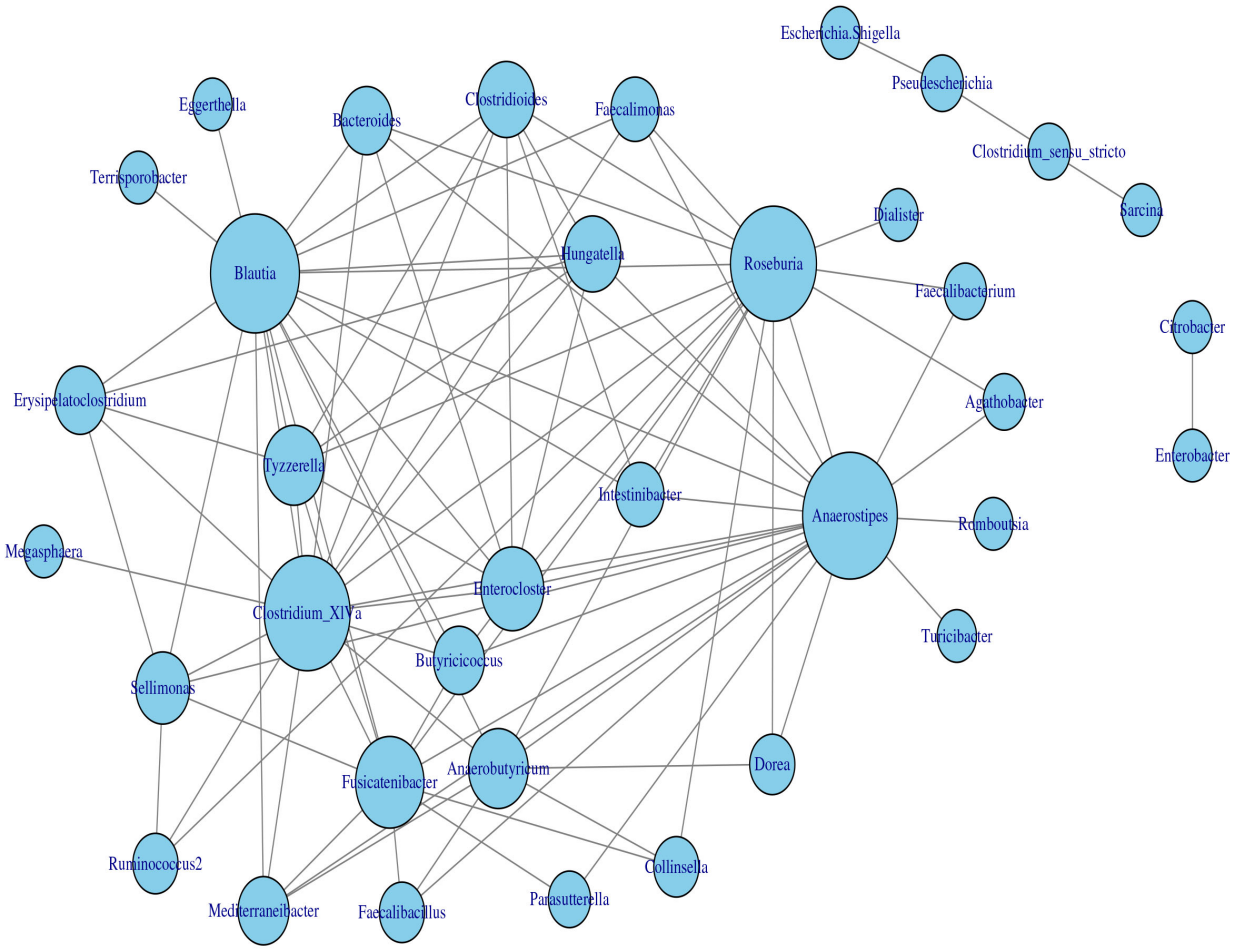
Microbial interaction network generated with the homogeneous LGLASSO based on the gut microbiome abundance data in Section 3.2.

The application of the homogeneous model in Section 2.2 to the transformed data yielded the estimated network, which is displayed in **Figure 7**. Based on the modularity maximization algorithm (Newman, 2006; Blondel et al., 2008), five communities were identified in the estimated network, which is listed in **Table 2**.

**Table 2:** Five communities selected by maximizing the modularity of the estimated MIN in **Figure 7**.

C1	Escherichia.Shigella, Clostridium.sensu.stricto, Sarcina, Pseudescherichia
C2	Blautia, Erysipelatoclostridium, Bacteroides, Tyzzerella, Megasphaera, Intestinibacter, Enterocloster, Hungatella, Terrisporobacter, Clostridioides, Clostridium.XlVa, Butyricicoccus
C3	Anaerostipes, Fusicatenibacter, Agathobacter, Faecalibacterium, Dorea, Collinsella, Faecalimonas, Mediterraneibacter, Romboutsia, Faecalibacillus, Anaerobutyricum, Roseb Parasutterella, Dialister, Turicibacter
C4	Enterobacter, Citrobacter
C5	Sellimonas, Ruminococcus2

**Figure 8:**
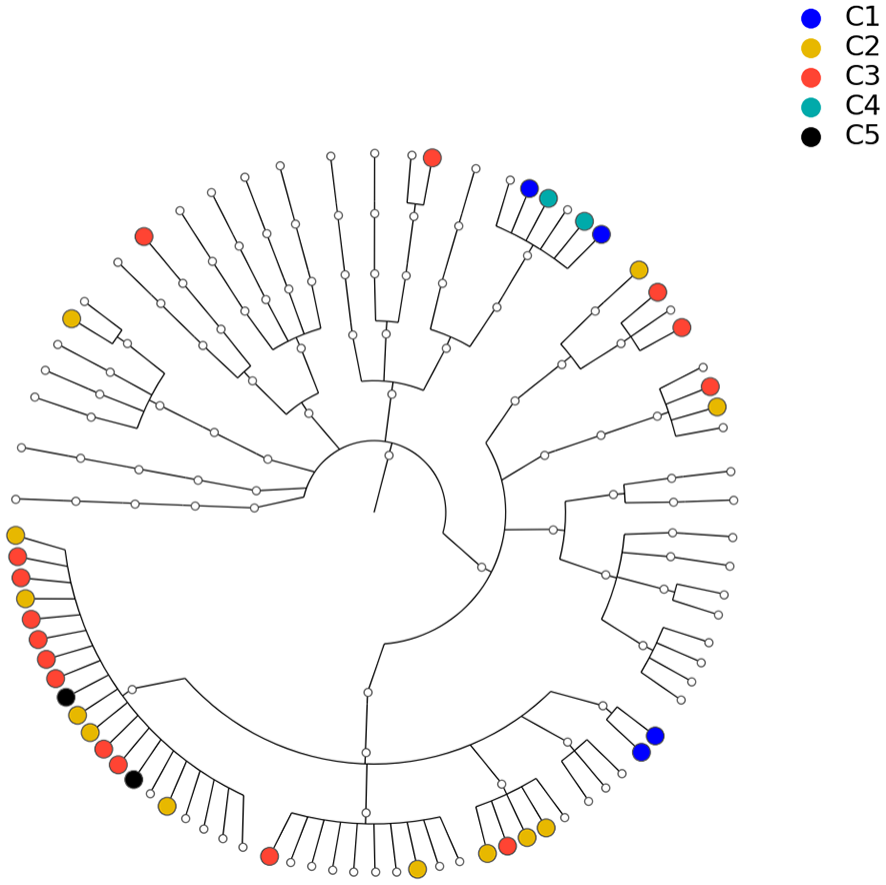
Phylogenetic tree for the 82 microbial taxa in Section 3.2. Dots with different colors correspond to different communities listed in **Table 2**.

To show that the estimated network can reveal the true structure of the underlying network, we investigated the relationship between the estimated network and the phylogenetic tree of the 82 taxa (the composite network was excluded here). The phylogenetic tree constructed from the same data set is presented in **Figure 8** and demonstrates the evolutionary relationship among these taxa. Our underlying hypothesis is that microbial taxa that are closer in terms of evolutionary history also have more/stronger interactions in the human body. To validate this hypothesis, the null hypothesis is set as follows: the estimated network in **Figure 7** is independent of the phylogenetic tree in **Figure 8**. Correlation between the estimated network and phylogenetic tree is employed to test this hypothesis. In particular, we computed the distances between two taxa in the estimated network and the phylogenetic tree. Here, the distance between taxa A and B is defined as the length of the shortest path from A to B in the estimated network (phylogenetic tree). If no paths exist between two taxa in the network (phylogenetic tree), that pair will be excluded from the following computations. Let *d*_1_ and *d*_2_ be the distance vectors for all possible pairs of taxa from the estimated network and phylogenetic tree, respectively. The correlation between *d*_1_ and *d*_2_ is used to measure the relatedness between the network and phylogenetic tree. This correlation was determined to be *r*_0 =_ 0.333 for the tuning parameter selected by the EBIC.

To understand the significance of *r*0 against the null hypothesis, we use the permutation method to estimate the null distribution. Specifically, we keep the structure of the estimated network unchanged and permute the order of the 82 taxa *m* **=** 5000 times on the estimated network. $Let\;d_{1}^{\left(i\right)}\left(i=1,\cdots ,5000\right)$ be the distance vectors of the network for the *i*th permutation. Then, the correlations $r^{\left(i\right)}=cor\left(d_{1}^{\left(i\right)},d_{2}\right),i=1,\cdots ,5000$ , which collectively depict the null distribution, can be derived. Given *r* ^(^ *^i^* ^)^, the p value of *r*_0_ is smaller than 1/5000, which means that the correlation between the estimated network and phylogenetic tree is statistically significant, i.e., the data support the hypothesis that microbial taxa with closer evolution histories tend to have more/stronger interactions.

It should be noted that some related findings have been reported in the literature. In (Chaffron et al., 2010), the authors performed a global meta-analysis of previously sampled microbial lineages in the environment. They found that genomes from coexisting taxa tended to be more similar than expected by chance, both with respect to pathway content and genome size. The studies in (Eiler et al., 2012) also revealed that ecological coherence is often dependent on taxonomic relatedness. These studies employed coefficient-based methods such as Fisher’s exact test to infer the interaction of taxa. This can lead to a misleading conclusion. It is known that the correlation between two taxa, A and B, may be induced by their correlation with a third taxon C, even though A and B are independent if C is fixed. In the current study, the interaction between the taxa is defined based on the conditional correlation coefficient, which by its definition eliminates the possible spurious correlation between taxa A and B induced by taxon C. Therefore, by using abundance instead of cooccurrence information and boosted by the proposed methods, we reach a more convincing and robust conclusion than existing ones in the literature.

**Figure 9:**
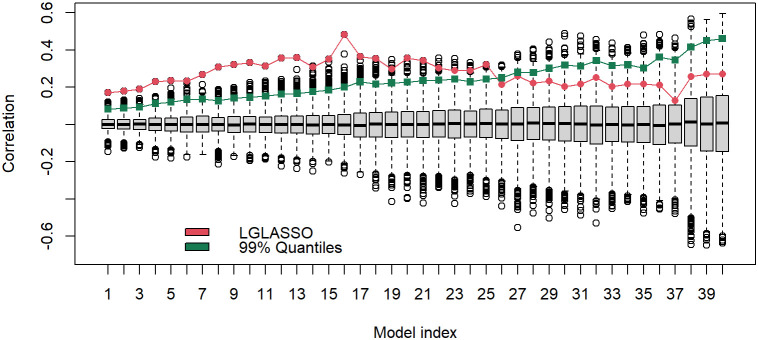
Boxplots of correlations between the networks and phylogenetic tree that correspond to, from left to right, the tuning parameters *λ* increasing from 7 to 13. For a given boxplot, the red dot represents the correlations from the original estimated networks, and the black dots represent correlations computed from 5000 permutations of the estimated network. The green dot represents the 1% upper quantile of the 5000 permutation correlations.

The results above are derived with the fixed tuning parameter selected by the EBIC. The correlations between the estimated MIN and phylogenetic tree are actually robust with respect to the tuning parameter. To demonstrate this point, let us consider the networks generated from other tuning parameter choices. Specifically, for each of the 40 tuning parameters ranging from *λ* **=** 7 to 13, the same homogeneous model and the permutation test are carried out. The estimated and permuted correlations are displayed in **Figure 9** in the form of a boxplot. From left to right, the boxplots in **Figure 9** correspond to the tuning parameters increasing from 7 to 13. The dots linked by the line represent the correlations between the estimated MINs and the phylogenetic tree, while others correspond to the correlations computed from the permuted MINs. The most prominent feature of **Figure 9** is that for all 40 cases, the observed correlations between the MINs and the phylogenetic tree are positive; in the first half of the boxplots, the estimated correlations are also significant. It should be noted that even though the estimated correlations in the second half of the boxplots do not appear significant, it does not mean that the edges in these estimated networks do not reflect the true structure. Instead, the insignificance may stem from the fact that the second half of the networks are sparser. A sparse network will generate shorter vectors *d*_1_ *, d*_2_, which in turn increase the variability of the correlation estimates.

**Figure 10:**
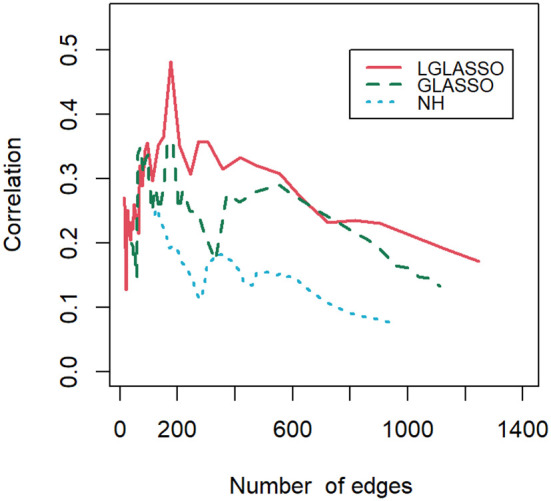
Correlations between the phylogenetic tree and networks selected by the LGLASSO, GLASSO, and NH methods for a sequence of tuning parameters.

Finally, let us compare the results of the proposed algorithm with those of the SPIECEASI algorithm in (Kurtz et al., 2015). Though in the original form of the SPIEC-EASI algorithm, the centered log-ratio transformation was employed for the relative abundances of the taxa, we use the additive log-ratio transformation here. Note that the SPIEC-EASI algorithm includes both graphical LASSO and neighborhood algorithms. We restrict ourselves to networks with edges between 20 and 1300, which we believe cover all biologically meaningful cases. First, we compute the solution path for each algorithm. For the networks on each solution path, their correlations with the phylogenetic tree are calculated. **Figure 10** displays the correlations for the three solution paths corresponding to the three algorithms. It is evident that for most of the solution paths, the networks generated through the proposed algorithm have higher correlations with the phylogenetic tree than those generated through the SPIEC-EASI algorithms. The correlations at the beginning parts of the three paths appear to be comparable. We attribute this to the fact that the networks at the beginning parts are much sparser, leading to a smaller sample size when computing the correlations. A smaller sample size can blur the comparison of different algorithms, as shown in **Figure 9**. In other words, if we assume that the phylogenetic tree represents the true structure of the MIN, then the proposed algorithms have greater power in the identification of the MIN than that of the SPIEC-EASI algorithm.

## 4 Discussion

Identifying microbial interaction networks is critical for understanding the causal relationship among taxa. However, it remains a challenging problem since observations of the microbiome have many distinct features, such as high dimensionality, zero inflation, and composition. In this study, we study network identification based on irregularly spaced longitudinal 16S rRNA gene sequencing data. For microbial abundance data, the correlations between different time points are typically omitted in practice due to technical difficulties. In this study, a model named SGGM is proposed to characterize the correlations in the longitudinal microbial abundance data. Efficient inference algorithms for estimating microbial interaction networks are devised based on the SGGM. Through the use of simulated data, our model and algorithms show that they have more power to identify microbial interaction networks than conventional methods, where the correlations are just omitted. Furthermore, the algorithms demonstrate their robustness when the data do not follow the SGGM strictly, e.g., heterogeneous microbial communities and zero inflation. The proposed method is employed to study the microbiomes from a cohort with cystic fibrosis disease. The relationship between the microbial interaction networks and the phylogenetic tree is revealed, strengthening previous literature results. It is also necessary to highlight the limitations of SGGM and the related LGLASSO algorithms. First, SGGM only models the stationary process, i.e., the microbial correlation structure remains the same during the data collection process. This may or may not be a valid assumption for a specific situation. For example, the subjects may get vaccinated during the data collection period, which may affect how the constituent microbes interact with each other. If this is the case, SGGM should not be used. Second, SGGM assumes a constant dampening rate *τ* for all the taxa in the microbiome within the same subject. We only studied the robustness of the LGLASS algorithms with respect to *τ* under very simple cases, i.e., two independent sub-microbial communities with different dampening rates. In reality, things can get very involved. For example, the whole community may have multiple sub-communities, and each of them has its own dampening rate, e.g., community A evolves with a high frequency, community B evolves with a medium frequency, community C evolves with a low frequency, and communities A, B, and C are related to each other in some way. In such cases, it should be cautious to use SGGM to identify the underlying network. Some form of cross-validation is recommended in such situations.

## Data availability statement

The original contributions presented in the study are included in the article/supplementary material. Further inquiries can be directed to the corresponding author. The proposed algorithms are available in the R package lglasso, which can be accessed at https://github.com/jiezhou-2/lglasso. The code for reproducing the simulation results in Section 3.1 can be found at https://jiezhou-2.github.io/lglasso_data_analysis/index.html. For access to the cystic fibrosis data used in Section 3.2, please contact the corresponding author at Anne.G.Hoen@dartmouth.edu.

## Author contributions

JZ: Conceptualization, Formal analysis, Investigation, Methodology, Software, Validation, Visualization, Writing – original draft, Writing – review & editing. JG: Conceptualization, Formal analysis, Funding acquisition, Investigation, Methodology, Supervision, Writing – review & editing. WV: Conceptualization, Methodology, Writing – review & editing. HC: Investigation, Writing – review & editing. SL: Writing – review & editing. JM: Data curation, Funding acquisition, Project administration, Writing – review & editing. MC: Conceptualization, Resources, Writing – review & editing. AH: Conceptualization, Formal analysis, Funding acquisition, Investigation, Methodology, Project administration, Supervision, Writing – original draft, Writing – review & editing.
